# Comprehensive proteomic analysis of exosomes derived from human bone marrow, adipose tissue, and umbilical cord mesenchymal stem cells

**DOI:** 10.1186/s13287-020-02032-8

**Published:** 2020-11-27

**Authors:** Zheng-gang Wang, Zhi-yi He, Shuang Liang, Qing Yang, Peng Cheng, An-min Chen

**Affiliations:** Department of Orthopedics, Tongji Hospital, Tongji Medical College, Huazhong University of Science and Technology, Wuhan, Hubei Province 430030 People’s Republic of China

**Keywords:** Mesenchymal stem cells, Exosomes, Proteomics, Extracellular vesicles, Stem cell-based therapy

## Abstract

**Background:**

Mesenchymal stem cell (MSC)-derived exosomes have shown comprehensive application prospects over the years. Despite performing similar functions, exosomes from different origins present heterogeneous characteristics and components; however, the relative study remains scarce. Lacking of a valuable reference, researchers select source cells for exosome studies mainly based on accessibility and personal preference.

**Methods:**

In this study, exosomes secreted by MSCs derived from different tissues were isolated, by ultracentrifugation, and proteomics analysis was performed. A total of 1014 proteins were detected using a label-free method.

**Results:**

Bioinformatics analysis revealed their shared function in the extracellular matrix receptor. Bone marrow MSC-derived exosomes showed superior regeneration ability, and adipose tissue MSC-derived exosomes played a significant role in immune regulation, whereas umbilical cord MSC-derived exosomes were more prominent in tissue damage repair.

**Conclusions:**

This study systematically and comprehensively analyzes the human MSC-derived exosomes via proteomics, which reveals their potential applications in different fields, so as to provide a reference for researchers to select optimal source cells in future exosome-related studies.

**Supplementary Information:**

The online version contains supplementary material available at 10.1186/s13287-020-02032-8.

## Background

In recent years, mesenchymal stem cells (MSCs) have been widely used in clinical treatment owing to their multi-differentiation potential [1, 2]. These cells play a significant role in immune regulation [3] and angiogenesis [4], especially in the heart, bone, cartilage, nerves, and skin [5–7]. There are many tissue-derived MSCs that have been used in therapeutic research, such as the bone marrow (BM), adipose tissue (AT), fetal lung, dental pulp, and umbilical cord (UC) [8, 9]. Paracrine secretion, particularly exosomes, is one of the main mechanisms of therapeutic action of MSCs [10], which has received much attention for its important roles in cellular communication and regenerative medicine.

Exosomes are lipid bilayer vesicles of 30–200 nm in diameter originating from endocytosis [11], which are secreted by multiple cells and appear in the supernatant of cell cultures or body fluids, such as the blood, saliva, urine, breast milk, cerebrospinal fluid, bile, and lymph [11–13]. Many types of molecules, including proteins, lipids, and nucleic acids, are present in exosomes [11]. When exosomes are absorbed by other cells, these molecules are transferred to the recipient cells and affect their physiological function, thus acting as intercellular communication vehicles and mediating many physiological and pathological processes [2, 14]. Studies have described several limitations to the direct use of MSCs, such as low survival rate, immunological rejection, and safety, and ethical issues [15–17]. Nevertheless, exosomes represent a safe and effective stem cell-free alternative therapeutic modality [18–20].

Previous studies have shown that MSC-derived from different tissues, as well as their secretome, have significant differences [21, 22]. As important components of the secretome, exosomes from different sources deserve further investigation. Most studies on exosomes have focused on RNA molecules (miRNA, lncRNA, and circRNA) [23–25]. However, MSC-derived exosomes are likely to play functional roles because of their proteins rather than RNAs, owing to their biologically relevant concentration, biochemical functionality, and potential to elicit an appropriate timely biochemical response [26].

Presently, exosomes derived from BM-, AT-, and UC-MSCs have gained the most attention. Therefore, in the present study, these three types of exosomes were selected for further investigation (SF. 1), and their morphology and surface markers were analyzed. Using a label-free method and bioinformatics tools, the systematic proteomic characteristics of exosomes were explored (SF. 1). This study aimed to provide novel insights for future human MSC-derived exosome research and treatment selection.

## Methods

### Culture and identification of MSCs

MSCs derived from different tissues (BM, AT, and UC) were obtained from Cyagen Biosciences (Suzhou, China). The cells were seeded in a specific culture medium (HUXMA-90011, HUXMD-90011, and HUXUC-90011, respectively; Cyagen Biosciences) and maintained at 37 °C and 5% CO_2_. The culture medium was changed every day. MSCs of the third passage were used in the following experiments.

To evaluate their multiple differentiation potential, MSCs were induced to differentiate by culturing in osteogenic, adipogenic, and chondrogenic differentiation medium (Cyagen), which confirmed by Alizarin red, Oil red O, and Alcian blue staining. MSC phenotypic analysis was performed using flow cytometry (BD Biosciences, San Jose, CA, USA), and the percentage of cells expressing mesenchymal markers—CD105, CD29, CD73, CD34, CD45, CD11b, and CD44 (Abcam, Cambridge, UK), was determined.

### Isolation and characterization of exosomes

Exosomes were isolated by ultracentrifugation as described previously [27]. Briefly, MSCs were swilled thrice with PBS and cultured in serum-free medium for 48 h. The supernatant was collected and used for gradient centrifugation at 300×*g* for 10 min, 2000×*g* for 10 min, and 10,000×*g* for 30 min to remove cells and debris. The supernatant was filtered through a 0.45-μm porous membrane and centrifuged at 120,000×*g* for 70 min, in an L-80XP ultracentrifuge equipped with a Ti70 rotor (Beckman Coulter, Brea, CA, USA). The pellet was resuspended in PBS and centrifuged again as described above. The exosomes were resuspended in PBS and stored at − 80 °C until further analysis.

Nanoparticle tracking analysis (NTA) through the NanoSight NS300 system (Malvern Instruments, Malvern, UK) was used to measure the concentration and size of exosomes. The morphological characteristics of exosomes were detected using transmission electron microscopy (TEM). The expression of surface markers including calnexin (ab13504), CD9 (ab254175), CD81 (ab219209), and Tsg101 (ab125011) (Abcam) on exosomes was analyzed by western blot.

### Protein extraction and trypsin digestion

The samples stored at − 80 °C were thawed and treated with lysis buffer (8 M urea, 1% protease inhibitor). After ultrasonic cracking and centrifugation, protein concentration was measured using a BCA kit (Tiangen Biotech, Beijing, China). Proteins were reduced with 5 mM dithiothreitol for 30 min at 56 °C. Iodoacetamide (11 mM) was used as a decontaminant at 24 °C in darkness, and 50 mM ammonium bicarbonate was added to reduce the urea concentration of dithiothreitol to 1 mM. Finally, the proteins were digested overnight with trypsin at 50:1 trypsin-to-protein mass ratio. The digested peptide was desalted using a C18 column.

### Liquid chromatography with tandem mass spectrometry (LC–MS/MS) analysis

The peptides were dissolved in mobile phase A solvent (0.1% (v/v) formic acid solution) and separated using a NanoElute ultra-high-performance liquid phase system from a C18 trap column to a C18 analytical column at a speed of 300 nL/min. Mobile phase B was an acetonitrile solution containing 0.1% formic acid. The peptides were subjected to NSI source followed by tandem mass spectrometry (MS/MS) in Q ExactiveTM Plus (Thermo Fisher Scientific, Waltham, MA, USA) coupled online to the UPLC. The electrospray voltage applied was 2.0 kV. The m/z scan range was 350 to 1800 for a full scan, and intact peptides were detected in the Orbitrap at a resolution of 70,000.

### Bioinformatics analysis

Maxquant software package (v1.6.6.0) was used to retrieve the secondary MS data, and the Swiss-Prot_Human data was used as reference (20,600 proteins; Proteome ID: UP000005640). The sequences of the identified proteins were mapped according to their Gene Ontology (GO), to determine their biological and functional properties, using InterProScan (v.5.14-53.0, http://www.ebi.ac.uk/interpro/). Protein-enriched pathways assessment was performed by the Kyoto Encyclopedia of Genes and Genomes (KEGG) database and the KAAS tool (v.2.0, http://www.genome.jp/kaas-bin/kaas_main). The identified proteins were compared with exosome available data from ExoCarta database (http://www.exocarta.org).

### Statistical analysis

All experiments were performed three times. Principal component analysis (PCA), relative standard deviation, and Pearson’s correlation coefficient were used to evaluate the quantitative repeatability of proteins. GraphPad Prism 7.0 (GraphPad Software, San Diego, CA, USA) was used for all statistical analyses. Results with a *P* value < 0.05 were considered statistically significant.

## Results

### Characteristics of MSCs and exosomes

**Fig. 1:**
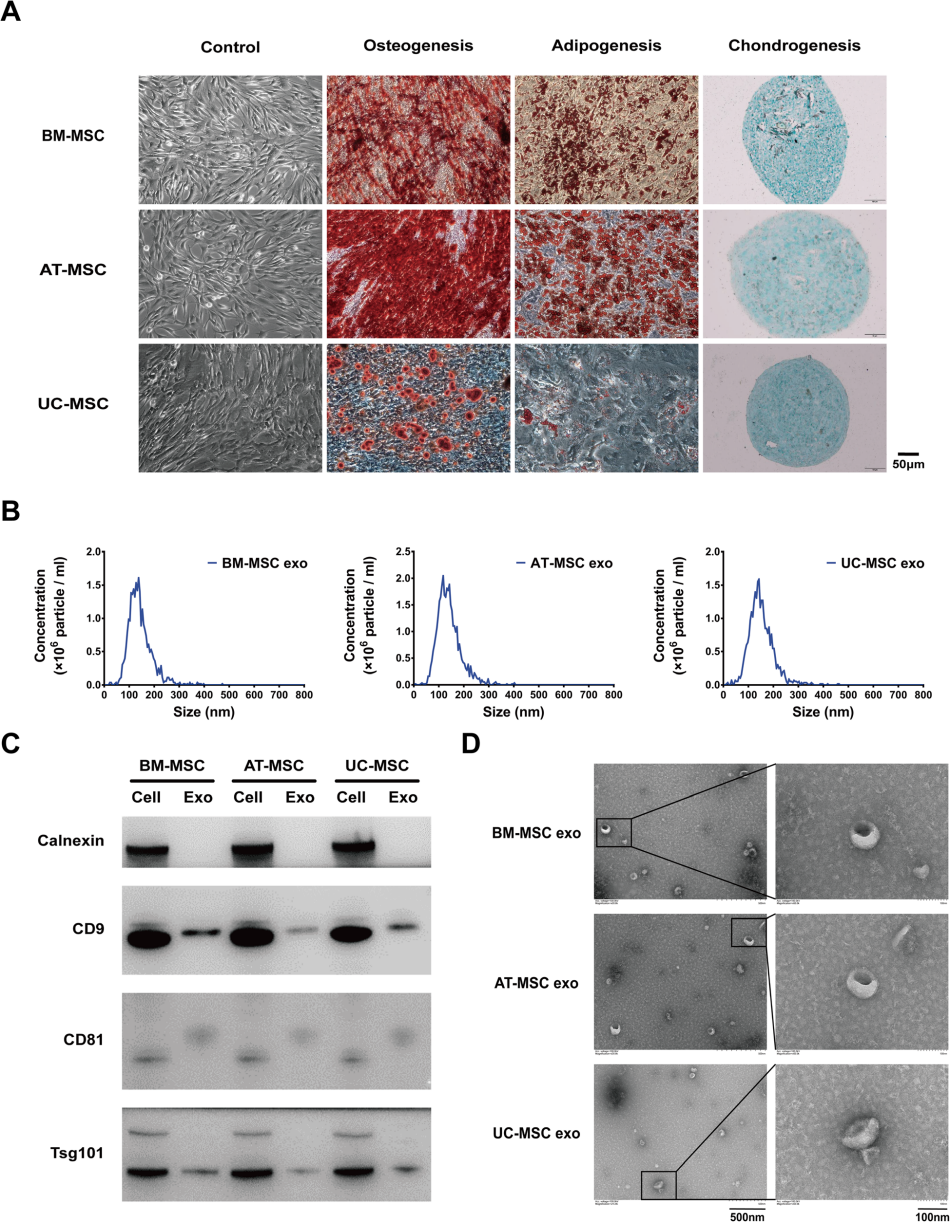
Quality control of MSCs and exosomes. **a** MSCs were stained with Alizarin red, Oil red O, and Alcian blue to confirm their differentiation into osteogenesis, adipogenesis, and chondrogenesis. (Scale bar 50 μm and 100 μm). **b** Size distribution of exosomes measured by NTA. **c** The MSCs and exosomes expression levels of Calnexin, CD9, CD81, and TSG101 were detected via western blot analysis. **d** TEM images of exosomes derived from MSCs. (Scale bar 100 nm, 500 nm)

The MSCs showed good morphology and excellent proliferation rates. Results of flow cytometry showed that the MSCs were positive for CD105, CD29, CD73, and CD44, and negative for IgG1, IgG2b, CD11b, CD34, and CD45 (SF. 2a-c). Cytochemical staining data further showed the differentiation potential of MSCs for osteogenesis, lipid formation, and cartilage formation, respectively (Fig. 1a). These results confirmed that the experimental cells had typical MSC characteristics.

The particle size distribution for exosomes was within 30–200 nm, with most exosomes (exo) having a 150-nm diameter. The concentration of AT-MSC exo was higher than that of BM-MSC exo or UC-MSC exo (Fig. 1b). Western blot analysis confirmed the presence of surface markers of the source cells and exosomes such as CD9, CD81, TSG101, and Calnexin, indicating that exosomes were representative (Fig. 1c). Typical cup-shaped vesicles were observed by TEM, and no differences in shape among the exosomes from the three different tissue sources were noted (Fig. 1d).

### Bioinformatics analyses of exosomes from different sources

Most of the peptides comprised 7–20 amino acids, meeting the quality control requirements for LC–MS/MS analysis (SF. 3a). The molecular weight of the protein was negatively correlated with the coverage (SF. 3b). The Pearson’s diagram demonstrated a high correlation among exosomes of the same cell origin. Among exosomes from different sources, the correlation between BM-MSC exo and UC-MSC exo was low (SF. 3c). The PCA diagram indicated good repeatability (SF. 3d).

**Fig. 2:**
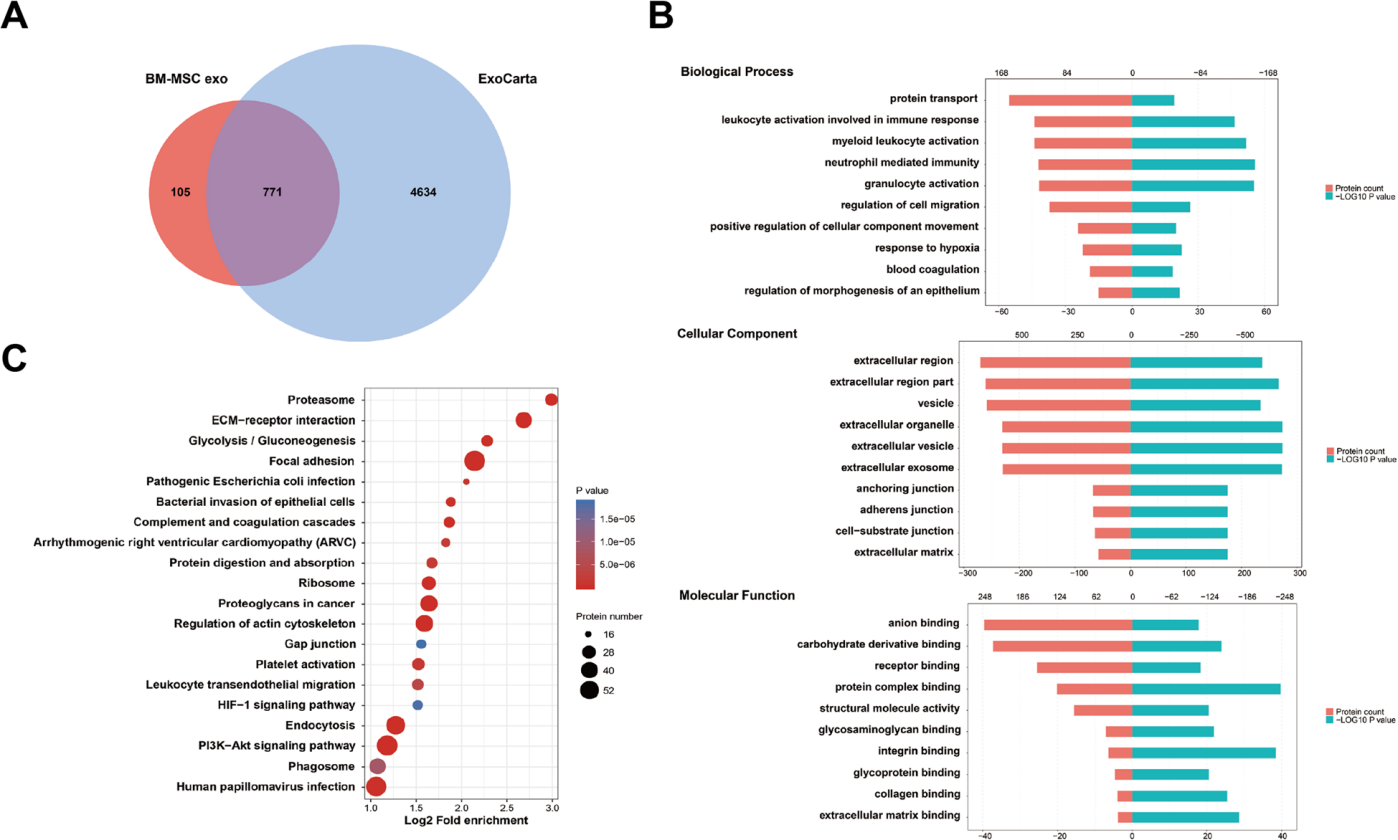
Bioinformatics analysis of BM-MSC exo. **a** Venn diagram of BM-MSC-derived exosomes against ExoCarta. **b** GO analysis of BM-MSC exo. **c** KEGG analysis of BM-MSC exo

**Fig. 3:**
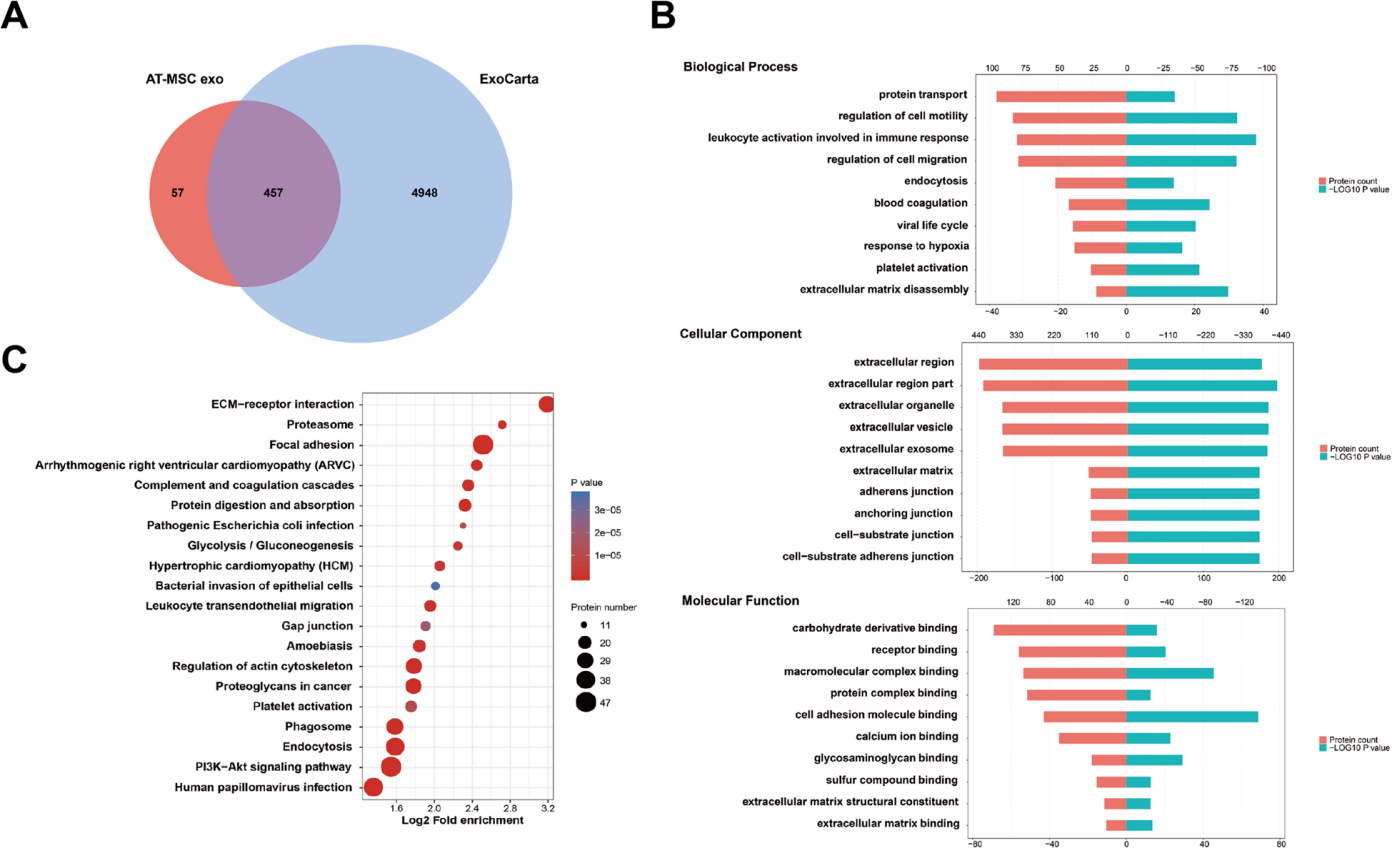
Bioinformatics analysis of AT-MSC exo. **a** Venn diagram of AT-MSC-derived exosomes against ExoCarta. **b** GO analysis of AT-MSC exo. **c** KEGG analysis of AT-MSC exo

**Fig. 4:**
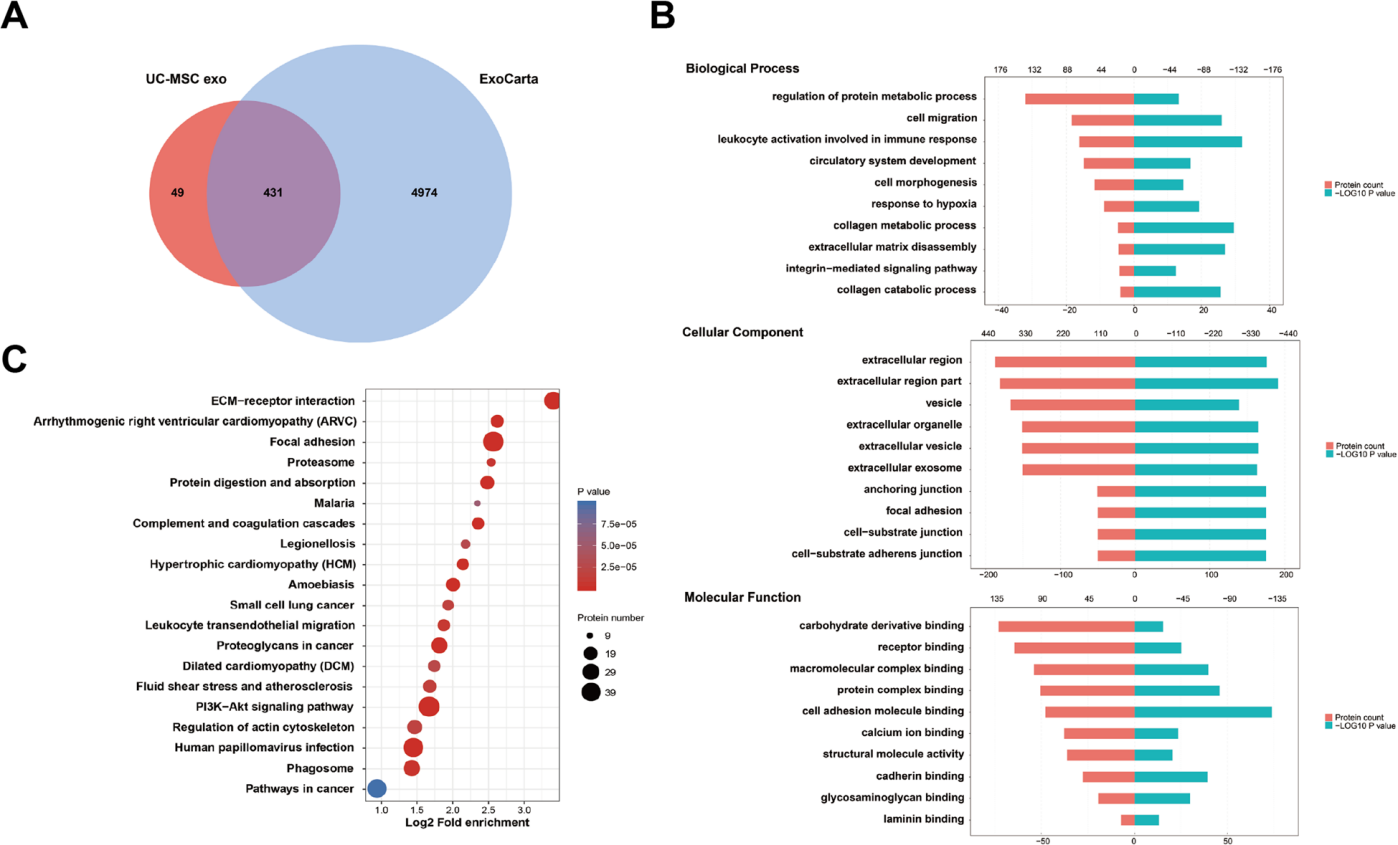
Bioinformatics analysis of UC-MSC exo. **a** Venn diagram of UC-MSC-derived exosomes against ExoCarta. **b** GO analysis of UC-MSC exo. **c** KEGG analysis of UC-MSC exo

A detailed proteomic analysis of the differently sourced-exosomes revealed 771, 457, and 431 proteins in BM-MSC exo, AT-MSC exo, and UC-MSC exo, respectively. Almost 90% of proteins in the three types of exosomes matched ExoCarta, indicating that the results were significantly reliable, and their unique proteins enriched the existing exosome library (Figs. 2a, 3a, and 4a). The GO analysis grouped the results as per biological process, cellular component, and molecular function. In the cellular component, the identified proteins were significantly enriched in extracellular region and cytoplasm region (Fig. 2b, 3b, and 4b). In terms of biological process, BM-MSC exo proteins were mainly involved in granulocyte activation and regulation of cell migration, whereas AT-MSC exo and UC-MSC exo proteins were enriched in leukocyte activation involved in immune response. In addition, UC-MSC exo proteins were also enriched in collagen metabolic process. As for molecular function, AT-MSC exo and UC-MSC exo proteins were both significantly enriched in cell adhesion molecule binding, whereas BM-MSC exo proteins were mostly involved in protein complex binding and integrin binding. The KEGG pathway analysis showed that all three exosome proteins were enriched in extracellular matrix (ECM)-receptor interaction, with BM-MSC exo proteins being mainly enriched in proteasome (Figs. 2c, 3c and 4c).

### Horizontal bioinformatics analysis of the three sources of exosomes

**Fig. 5:**
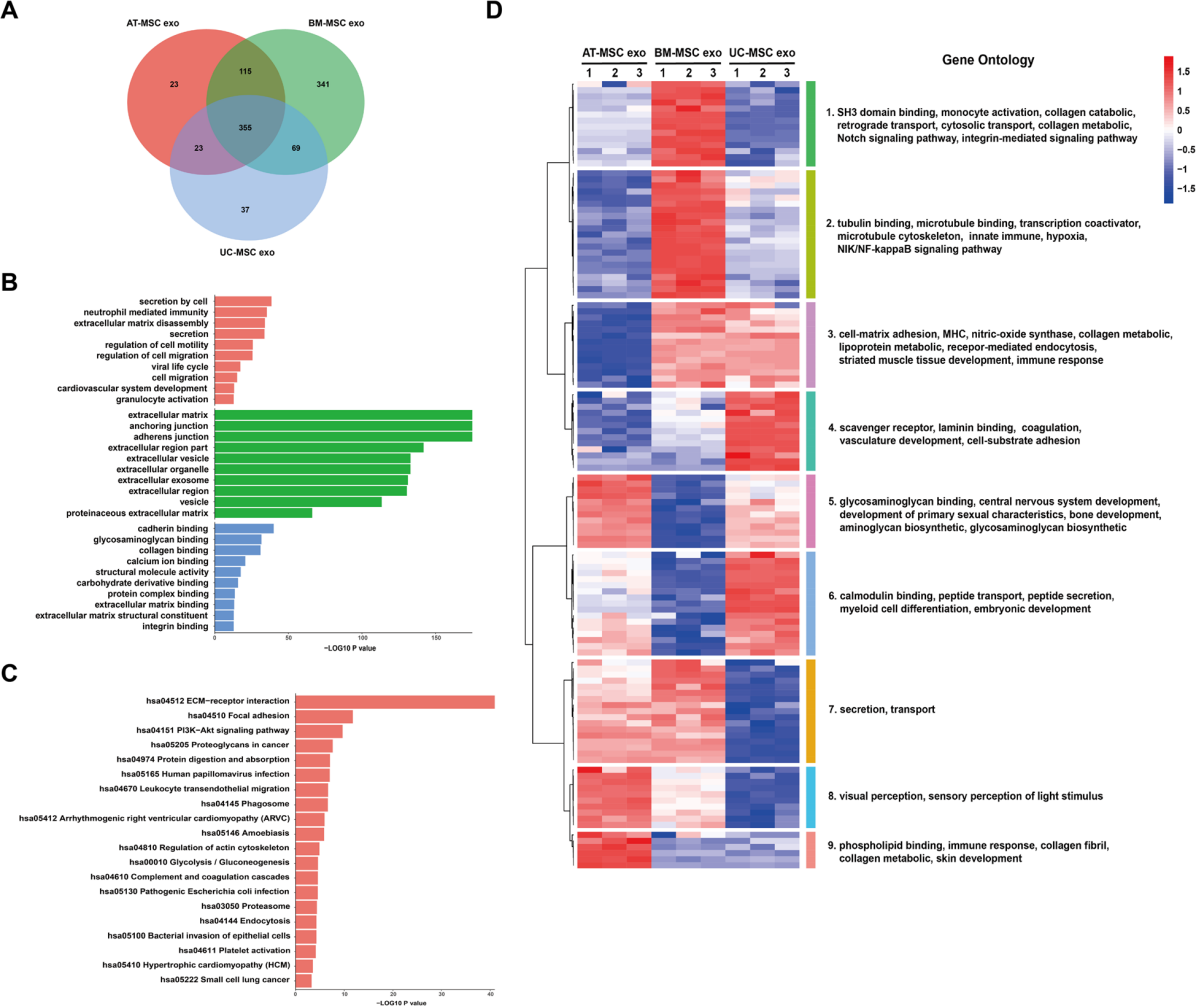
Horizontal bioinformatics analysis of three sources of exosome proteins. **a** Venn diagrams of detected proteins in exosomes derived from human MSCs. **b** GO analysis of the shared proteins among three sources of MSCs. (Red: biological process, green: cellular component, blue: molecular function.) **c** KEGG analysis of the shared proteins among three sources of MSCs. **d** Heat map of the protein level of shared proteins among three sources of MSCs. Clusters are assembled by GO analysis. The significantly enriched items are shown on the right

Comparison of the three types of exosomes using a Venn diagram revealed 355 common proteins, and 341, 23, and 37proteins unique to BM-MSC exo, AT-MSC exo, and UC-MSC exo, respectively (Fig. 5a). Further GO analysis of the shared proteins showed that most of the proteins in the cellular component were concentrated in the extracellular matrix, anchoring junction, and adherent junction. Furthermore, proteins were enriched in secretion by cells at the biological process level and enriched in cadherin binding of molecular function (Fig. 5b). Moreover, in the KEGG pathway analysis, the shared proteins were significantly enriched in ECM-receptor interaction (Fig. 5c). According to the heat map, proteins in clusters 1 and 2, which comprised proteins involved in transport and pathways, were enriched in BM-MSC exo. Proteins in cluster 3, metabolic and immune proteins, were depleted in AT-MSC exo. Receptor and binding proteins (cluster 4) were enriched in UC-MSC exo, whereas BM-MSC exo was depleted of proteins involved in tissue development (clusters 5 and 6). Proteins in clusters 7 and 8, secretion and transport proteins, were depleted in UC-MSC exo. Proteins in cluster 9, proteins of immune and metabolic processes, were enriched in AT-MSC exo (Fig. 5d).

**Fig. 6:**
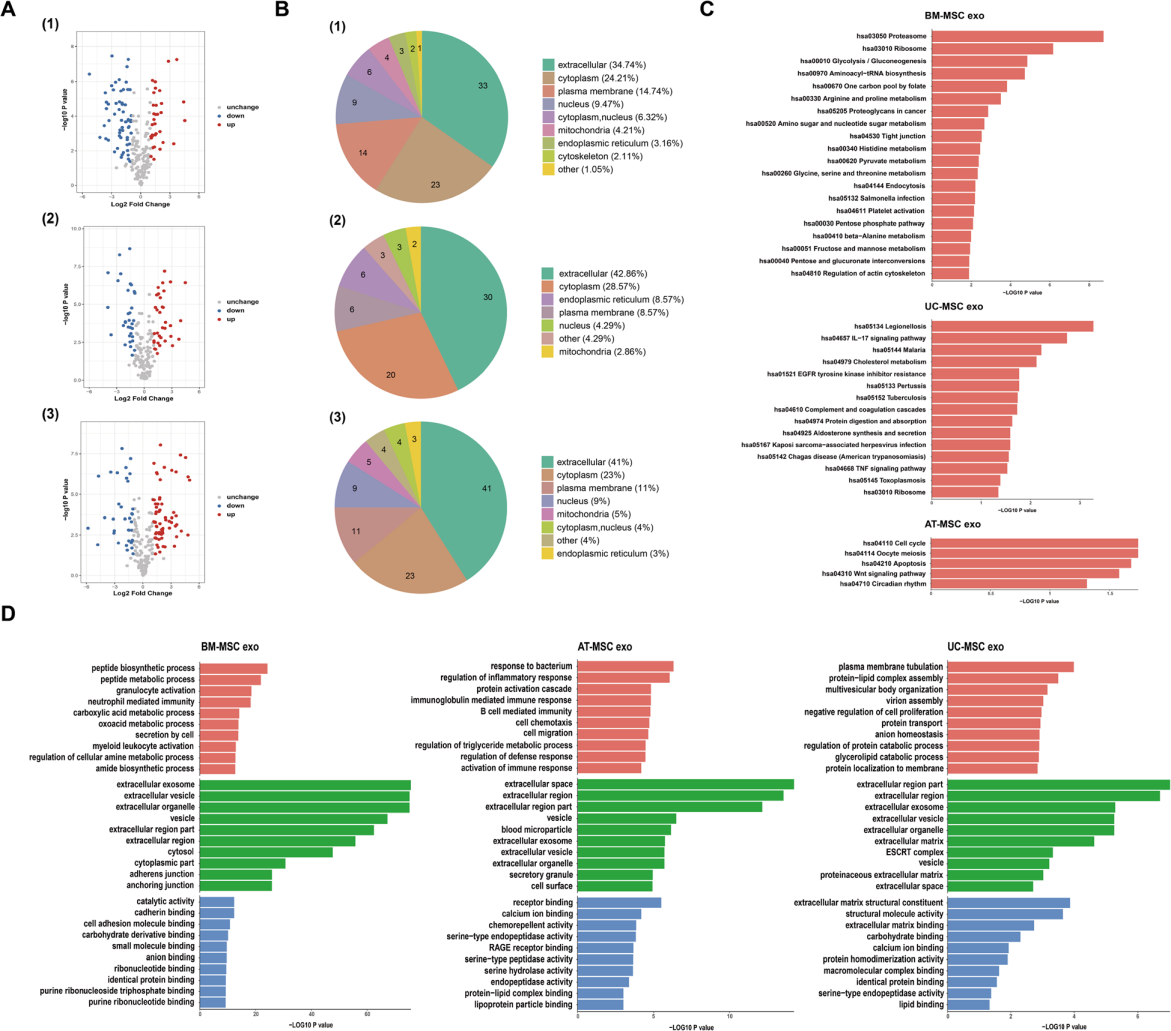
Bioinformatics analysis of the specific proteins detected in each source of exosomes. **a** The volcanogram of differential proteins for BM-MSC exo vs AT-MSC exo, BM-MSC exo vs UC-MSC exo, and AT-MSC exo vs UC-MSC exo. **b** The cytolocalization of differential proteins for BM-MSC exo vs AT-MSC exo, BM-MSC exo vs UC-MSC exo, and AT-MSC exo vs UC-MSC exo. **c** GO analysis of specific proteins for each source of exosomes. (Red: biological process, green: cellular component, blue: molecular function). **d** KEGG analysis of specific proteins for each source of exosomes

The volcanogram reflected the differential protein expression between every two groups (Fig. 6a). These differential proteins were mainly located in the extracellular region, followed by the cytoplasm (Fig. 6b). According to GO analysis, BM-MSC exo showed notable differences in terms of cellular components, when compared with AT-MSC exo and UC-MSC exo. Among them, the specific proteins of AT-MSC exo and UC-MSC exo were mostly enriched in the extracellular space and extracellular region, and BM-MSC exo was mostly enriched in the extracellular exosome and extracellular vesicle (Fig. 6c). In KEGG analysis, BM-MSC exo proteins were enriched in the proteasome, UC-MSC exo proteins were enriched in legionellosis, and AT-MSC exo proteins were enriched in cell cycle and oocyte meiosis (Fig. 6d).

### Differentially expressed membrane proteins in exosomes

**Fig. 7:**
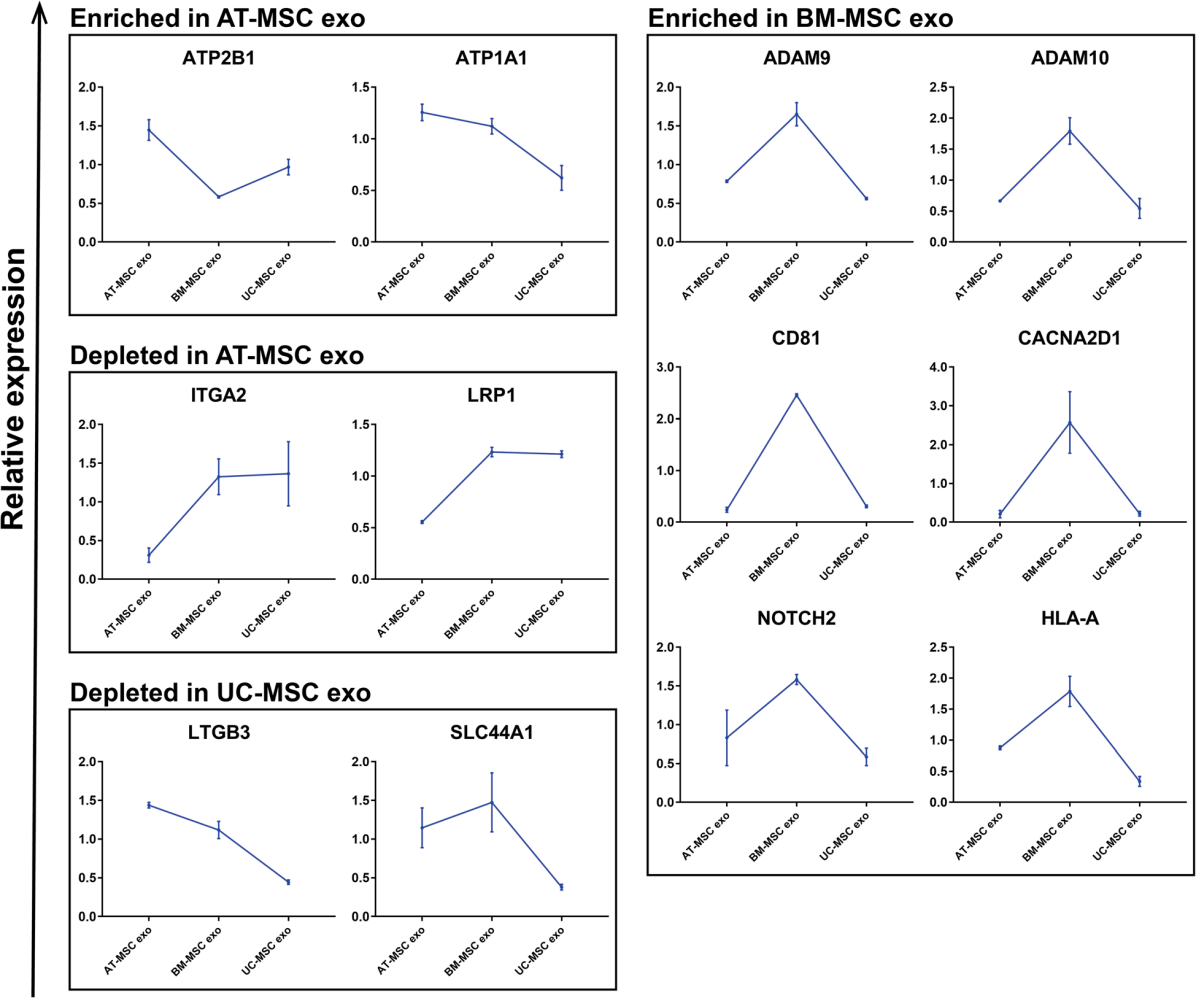
Membrane protein enrichment in exosomes depends on source cell type. Relative expression of each membrane protein within a sample is depicted on slop charts and membrane protein grouped according to their enrichment or depletion in AT-MSC exo, enrichment in BM-MSC exo, and depletion in UC-MSC exo

Lastly, the differentially expressed membrane proteins were analyzed in a line graph. Our analysis revealed that ATP2B1 and ATP1A1 showed high expression in AT-MSC exo, whereas ITGA2 and LRP1 showed low expression. LTGB3 and SLC44A1 showed low expression in UC-MSC exo. In contrast, ADAM9, ADAM10, CD81, CACNA2D1, NOTCH2, and HLA-A showed high expression in BM-MSC exo (Fig. 7).

## Discussion

MSCs and their secretome have been regarded as a significant in many research areas, such as regeneration, immunity, and inflammation [14]. Extracellular vesicles (EVs) are important components of the secretome. Based on their biological origin and physical dimensions, there are two EV subgroups in particular, namely, exosomes and microvesicles (MVs). Although exosomes and MVs have various proteins in common [28], there are some important differences between them. Previous studies have shown that proteins related to extracellular matrix, adherens junctions and integrins, and vesicle-mediated transport are enriched in exosomes, whereas proteins related to mitochondria, endoplasmic reticulum, and cytoskeleton are enriched in MVs [29, 30]. With the recent advances in the field of extracellular vesicles, the methods for the isolation and purification of exosomes are constantly optimized, meeting the rigor needed for comparative studies [31]. Therefore, there is an increasing interest in the study of exosomes.

Compared with MSC therapy, exosomes have the advantages of improved safety, easier preservation, fewer side effects, and lesser ethical issues [15, 16]. Exosomes function by transversal transfer of proteins, mRNAs, and miRNAs [11]. Disease status can change the exosome contents [32], and the composition of exosomes from various sources varies. Exosomes isolated from AT-derived MSCs contained four times higher levels of neutral lysins than those from BM-MSCs [33]. Moreover, the exosomes isolated from BM- and UC-derived MSCs showed significantly different in vivo effects on U87MG glioblastoma cells [34].

In previous studies, researchers have mainly considered the accessibility of exosomes and selected exosomes specifically related to their research fields, paying less attention to the differences between exosomes derived from different sources. Some studies have explored the proteomic profile of different MSCs [8] and compared the proteomics of secretomes [22]. We specifically focused on the exosome proteome and systematically compared the characteristics of exosomes derived from MSCs of three different tissue sources to provide a reference for the selection of exosomes.

Exosomes from BM-, AT-, and UC-MSCs were similar in morphology and immunophenotype. Furthermore, the shared proteins were concentrated in the extracellular matrix receptor, which suggests common properties between the exosomes. Their corresponding functions, such as migration, proliferation, adhesion, and apoptosis, have been shown in exosomes of different sources [34–36].

BM-MSC exo, as the first studied stem cell exosome, were mainly evaluated in the field of the bone and cartilage regeneration, and angiogenesis [37–39]. Zuo et al. showed that exosomes derived from irradiated BM-MSCs could prevent bone mass loss via the Wnt/β-catenin pathway [38]. In our study, proteins related to collagen, extracellular matrix, bone regeneration, and muscle regeneration were enriched, revealing the excellent potential of exosomes in regeneration medicine. For example, Notch2 was enriched in BM-MSC exo. Notch signaling is an evolutionarily conserved pathway that can mediate critical cell communication for embryonic development and tissue regeneration. In particular, Notch2 increases skeletal remodeling in osteoprogenitor cells [40]. In addition, previous studies have shown that exosomes can be absorbed by peripheral nerve endings and transported in reverse, as well as partake in neuroplasticity [41, 42]. Interestingly, we found that ADAM10 and CA2D1 showed high expression in BM-MSC exo. ADAM10 is involved in neurodevelopment, synaptic plasticity, and dendritic spine morphology, and it has been well studied as a therapeutic target for brain diseases [43, 44]. CA2D1, an early biomarker of vestibular schwannoma, is observed in response to spinal injury and neuropathic pain [45–47]. Moreover, Pires et al. indicated that the secretome of BMSC might be the most advantageous choice for a therapy designed to reduce oxidative stress [22]. Further studies are needed to confirm this possible modulation in neurological diseases.

AT-MSCs were found to hold the most significant secretory function among the three types of MSCs evaluated. Considering their easy availability and fewer ethical issues, AT-MSCs may present a good choice for mass production of exosomes. Previous studies described the role of AT-MSC exo in immune dysregulation, oxidative stress, and inflammation [48, 49]. Lee et al. showed that AT-MSCs had the ability to stably produce exosomes, and PRDX 1, 2, 4, and 6 exerted antioxidant activity to protect the kidney during acute injury [49]. Our data further confirmed that exosomes play a role in immunity primarily by the regulation of leukocyte-mediated immunity. In addition, based on GO analysis, AT-MSC exo may facilitate clotting, which is consistent with the findings of Chance et al. [50]. However, the clinical application for this aspect is still rare.

Studies on UC-MSC exo have shown that they play a significant role in tissue damage repair; however, the specific mechanism are yet to be elucidated [51]. Among various research directions, skin regeneration is a hot spot [52, 53]. Zhang et al. found that 14-3-3ζ of the UC-MSC exo could coordinate Wnt4 signaling by promoting YAP phosphorylation to repair damaged skin tissue; however, there is no evidence indicating whether exosome-delivered Wnt4 is superior to exosome-free Wnt4 [52]. Herein, it was found that PAI-1 protein was enriched in UC-MSC exo. PAI-1 is a single-stranded globular glycoprotein that plays a significant role in maintaining endothelial homeostasis and regulating fibrosis [54, 55]. During tissue damage, PAI-1 contributes to faster wound healing by inhibiting uPA/tPA/plasminogen and plasminogen-dependent MMP activity [54].

Although the lack of validation is a major limitation of this study, we comprehensively analyzed the proteomic profile of exosomes from MSCs of three tissue origins and compared their shared and unique proteins. Significant differences in proteins were found among BM-, AT-, and UC-derived MSC exosomes, and molecular targets for potential therapeutic purposes were identified.

## Conclusions

To the best of our knowledge, this is the first study to systematically and comprehensively analyze the human MSC-derived exosomes via proteomics. Our findings will provide helpful information for selecting optimal source cells for exosomes to improve research and therapeutic outcomes.

## Supplementary Information

**Additional file 1: SF. 1.** Workflow of exosome preparation and mass spectrometry. Human bone marrow (BM), umbilical cord (UC), and adipose tissue (AT)-derived MSCs were cultured, and exosome samples were obtained from the different cell line types by ultracentrifugation. Exosomes were subjected to proteomic analysis (nine samples, LC–MS/MS). Proteins were quantified using the label-free quantification method iBAQ.

**Additional file 2: SF. 2.** Flow cytometric analysis of cell immunophenotype of MSCs.

**Additional file 3: SF. 3.** Sample repeatability test and quality control of mass spectrometry. (a) The distribution of peptide length is showed in a histogram. (b) Protein mass and coverage distribution. (c) Pearson correlation coefficient between all samples is presented. (d) Protein quantitative principal component analysis results of all samples.

## Data Availability

All proteomics data have been deposited to the ProteomeXchange Consortium via the PRIDE partner repository with the dataset identifier PXD020948.

## Abbreviations

MSCs: Mesenchymal stem cells
BM: Bone marrow
AT: Adipose tissue
UC: Umbilical cord
NTA: Nanoparticle tracking analysis
TEM: Transmission electron microscopy
LC–MS/MS: Liquid chromatography with tandem mass spectrometry
GO: Gene Ontology
KEGG: Kyoto Encyclopedia of Genes and Genomes
exo: Exosomes
ECM: Extracellular matrix
