# Supplementary figures and images for: Comprehensive proteomic analysis of exosomes derived from human bone marrow, adipose tissue, and umbilical cord mesenchymal stem cells
Source: Stem Cell Res Ther. 2020 Nov 27;11:511. doi: 10.1186/s13287-020-02032-8 (PMC7694919; doi:10.1186/s13287-020-02032-8)

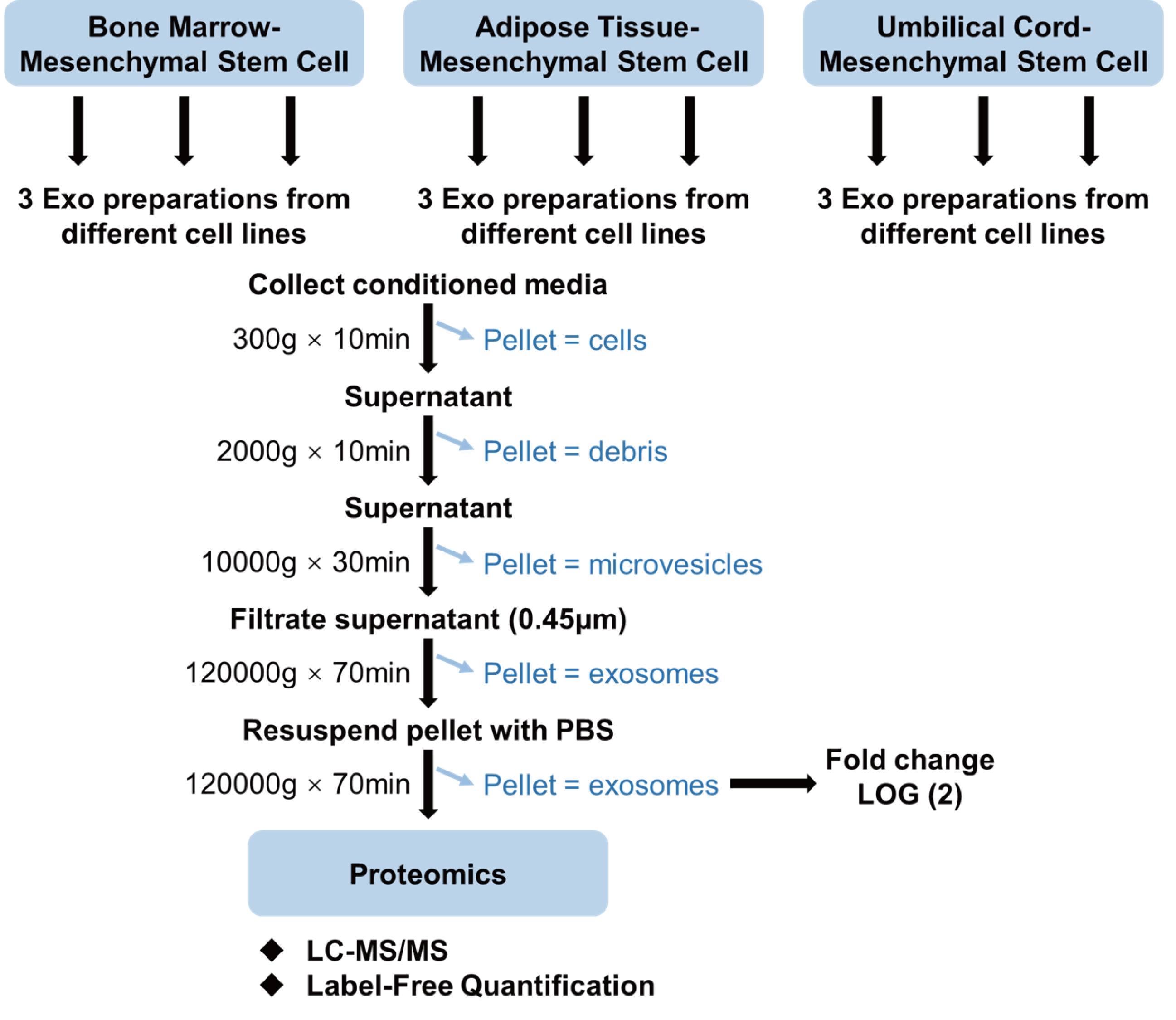

Supplement: Supplementary file 1 — Additional file 1: SF. 1. Workflow of exosome preparation and mass spectrometry. Human bone marrow (BM), umbilical cord (UC), and adipose tissue (AT)-derived MSCs were cultured, and exosome samples were obtained from the different cell line types by ultracentrifugation. Exosomes were subjected to proteomic analysis (nine samples, LC–MS/MS). Proteins were quantified using the label-free quantification method iBAQ. [file 13287_2020_2032_MOESM1_ESM.tif]

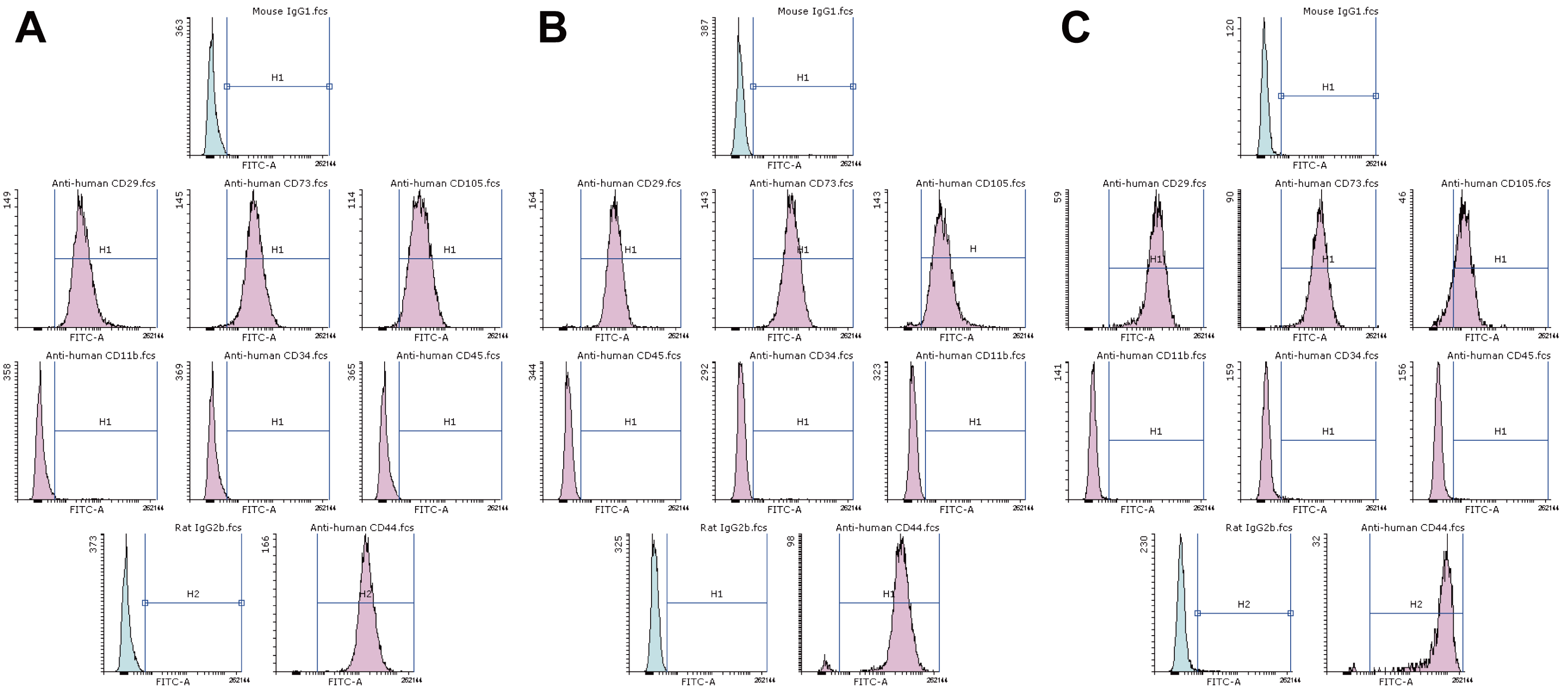

Supplement: Supplementary file 2 — Additional file 2: SF. 2. Flow cytometric analysis of cell immunophenotype of MSCs. [file 13287_2020_2032_MOESM2_ESM.tif]

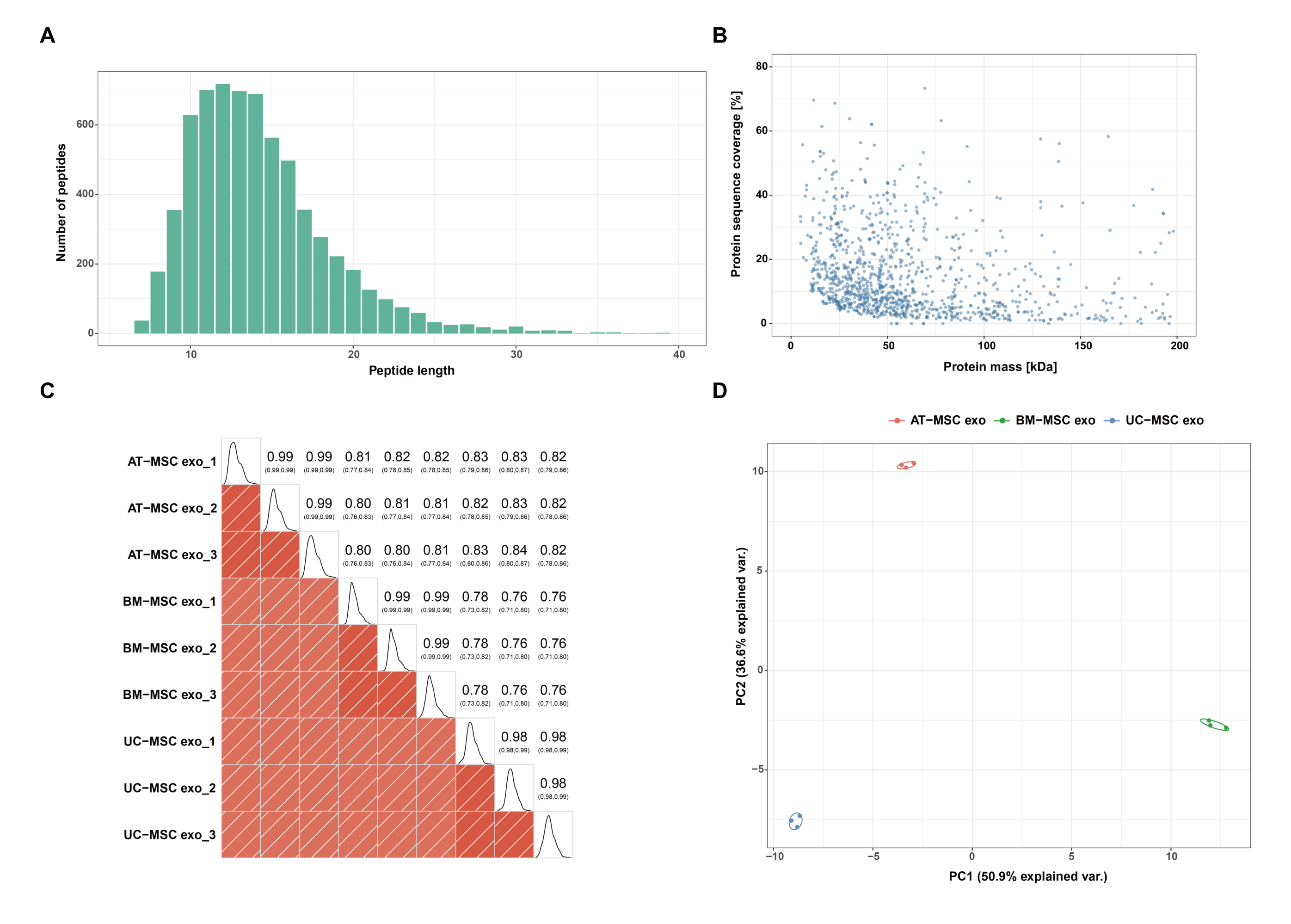

Supplement: Supplementary file 3 — Additional file 3: SF. 3. Sample repeatability test and quality control of mass spectrometry. (a) The distribution of peptide length is showed in a histogram. (b) Protein mass and coverage distribution. (c) Pearson correlation coefficient between all samples is presented. (d) Protein quantitative principal component analysis results of all samples. [file 13287_2020_2032_MOESM3_ESM.tif]
